# Evaluation of Rhomboid Intercostal Block in Video-Assisted Thoracic Surgery: Comparing Three Concentrations of Ropivacaine

**DOI:** 10.3389/fphar.2021.774859

**Published:** 2022-01-17

**Authors:** Wei Deng, Chen-Wei Jiang, Ke-jian Qian, Fen Liu

**Affiliations:** ^1^ Department of Critical Medicine, The First Affiliated Hospital of Nanchang University, Nanchang, China; ^2^ Medical Innovation Center, First Affiliated Hospital of Nanchang University, Nanchang, China; ^3^ Department of Anesthesiology and Pain Medicine, The Affiliated Hospital of Jiaxing University, Jiaxing, China

**Keywords:** rhomboid intercostal blocky, quality of recovery, video-assisted thoracoscopic surgery, anesthesia, analgesic

## Abstract

**Background:** Ultrasound-guided rhombic intercostal block (RIB) is a novel regional block that provides analgesia for patients who have received video-assisted thoracoscopic surgery (VATS). The anesthetic characteristics of ultrasound-guided RIB with different concentrations of ropivacaine are not known. This research primarily hypothesizes that ultrasound-guided RIB, given in combination with the same volume of different concentrations of ropivacaine, would improve the whole quality of recovery-40 (QoR-40) among patients with VATS.

**Approaches**: This double-blinded, single-center, prospective, and controlled trial randomized 100 patients undergoing VATS to receive RIB. One hundred patients who have received elective VATS and satisfied inclusion standards were fallen into four groups randomly: control group with no RIB and R_0.2%_, R_0.3%_, and R_0.4%_; they underwent common anesthesia plus the RIB with ropivacaine at 0.2%, 0.3%, and 0.4% in a volume of 30 ml.

**Outcomes:** Groups R_0.2%_, R_0.3%_, and R_0.4%_ displayed great diversities in the overall QoR-40 scores and QoR-40 dimensions (in addition to psychological support) by comparing with the control group (Group C) (*p* < 0.001 for all contrasts). Groups R_0.3%_ and R_0.4%_ displayed great diversities in the overall QoR-40 scores and QoR-40 dimensions (in addition to psychological support) by comparing with the R_0.2%_ group (*p* < 0.001 for all contrasts). The overall QoR-40 scores and QoR-40 dimensions [physical comfort (*p* = 0.585)] did not vary greatly between Groups R_0.3%_ and R_0.4%_ (*p* > 0.05 for all contrasts). Groups R_0.2%_, R_0.3%_, and R_0.4%_ showed significant differences in numerical rating scales (NRS) score region under the curve (AUC) at rest and on movement in 48 h when compared with the Group C (*p* < 0.001 for all contrasts). Groups R_0.3%_ and R_0.4%_ displayed great diversities in NRS score AUC at rest and on movement in 48 h when compared with the R_0.2%_ group (*p* < 0.001 for all contrasts). The NRS mark AUC at rest and, on movement in 48 h, did not vary greatly between the Group R_0.3%_ and R_0.4%_ (*p* > 0.05 for all contrasts).

**Conclusion:** In this study it was found that a dose of 0.3% ropivacaine is the best concentration for RIB for patients undergoing VATS. Through growing ropivacaine concentration, the analgesia of the RIB was not improved greatly.

**Clinicaltrials.gov Registration:** https://clinicaltrials.gov/, identifier ChiCTR2100046254.

## Introduction

Post-video-helped thoracoscopic surgery (VATS) pain is a serious and ongoing widespread concern (Jung et al., 2016; Rodriguez-Aldrete et al., 2016; Umari et al., 2018). Moderate to severe pain after VATS is associated with longer hospital stays, readmissions, low patient satisfaction, increased costs, decreased quality of life, and development of chronic pain (Bendixen et al., 2016; Wang et al., 2017; Sun et al., 2020). A variety of analgesic methods have been used to reduce the intensity of acute pain after VATS, including intravenous opioids, local anesthetic drug infiltration, intercostal nerve blocks, paravertebral blocks, and thoracic epidural blocks (Piccioni et al., 2018; Umari et al., 2018; Martin and Mehran, 2019; Bai et al., 2020; Dastan et al., 2020; Taketa et al., 2020). Opioids alone appear to be effective in controlling persistent pain but not episodic pain associated with cough and movement (Hah et al., 2019; Nobel et al., 2019). This would require higher plasma levels of these drugs, which would cause the resulting side effects of sedation and hypoventilation (Umari et al., 2018), weak analgesic effect of local anesthetic infiltration, and intercostal nerve block with short analgesic duration (Kaushal et al., 2019; Sheets et al., 2020). Aravertebral blocks and thoracic epidurals can cause total spinal anesthesia and parasympathetic symptoms, leading to hypotension, bradycardia, and even cardiac arrest (Yeung et al., 2016; Taketa et al., 2020).

The rhomboid intercostal block (RIB) is a novel kind of plane block illustrated by Elsharkawy et al. (2016) recently. They found that local anesthetic spreads across the interfascial plane between the intercostal muscles, penetrates deeply into the anterior serratus muscle, and extends through the rhomboid intercostal plane to the erector spinae; this fascial block has the most significant advantage, as it covers both dorsal rami and lateral cutaneous branches of the thoracic nerves (Altıparmak et al., 2019). Recent studies have shown that RIB can provide good analgesia after VATS and that its analgesic effect is also good compared to other nerve blocks (Adderley and Mullins, 1987; Ince et al., 2020; Deng et al., 2021a; Deng et al., 2021b; Jiang et al., 2021). However, the analgesic effects of different concentrations of RIB blockade after VATS have not been reported in clinical randomized controlled trials, and in order to enable patients to receive both adequate analgesia and reduce unnecessary adverse effects of local anesthetics, we compared the analgesic effect of different concentrations of ropivacaine RIB block after VATS. The experimental study by Deng et al. (2020) used 0.2%, 0.3%, and 0.4% concentrations of ropivacaine for thoracic nerve block and were safe and effective. However, the safety of using higher concentrations of 0.5% and 0.75% ropivacaine for thoracic nerve blocks remains controversial, so 0.2%, 0.3%, and 0.4% ropivacaine were chosen for RIB blocks for the safety of patients in this experiment.

As reported in previous studies, the quality of recovery-40 (QoR-40) provides a broad and valid evaluation on patients’ recovery quality after anesthesia and surgery, which can appropriately reflect the quality of postoperative recovery in a scope of clinical and study situations (Myles et al., 2000; Kim et al., 2018). Up to now, the analgesic effects of different concentrations of ropivacaine RIB after VATS were not evaluated by prospective researches with QoR-40.

In consideration of the gaps in scientific literature, this research primary aimed to compare the analgesic roles of 0.2, 0.3, and 0.4% ropivacaine after VATS by QOR-40 scores after 24 h. The secondary aim was to compare the need for 0.2, 0.3, and 0.4% ropivacaine RIB for the region under the receiver operating characteristic curve (AUC) of numerical rating scale (NRS) pain marks, postoperative opioid consumption, and rescue analgesia after VATS.

## Approaches

### Participants and Research Design

The research was a prospective, single-center, and randomized-controlled trial. This study was ethically approved by the Ethics Committee of the Affiliated Hospital of Jiaxing University (LS2021-KY-061), Jiaxing, China on April 16, 2021. The following principles summarized in the Declaration of Helsinki were performed. The registration of research protocol was made in the Chinese Clinical Trial Register (ChiCTR2100046254, links to registration documents: https://www.chictr.org.cn/edit.aspx?pid=126397&htm=4). The Chinese Clinical Trial was registered on May 12, 2021 (May 12, 2021), and patients were enrolled on May 14, 2021 (May 14, 2021). All patients who were screened and met the eligibility standards were invited to take part in the trial, and patients enrolled provided written informed consent. Patients were required to give consent on arrival at the operating room or if they were hospitalized at the night before the surgery. Inclusion standards were American Society of Anesthesiologists (ASA) grades 1–3, age 18–80 years, patients receiving general anesthesia for unilateral VATS, and no contraindications to peripheral regional anesthetic block. Exclusion standards were contraindication to local anesthesia, pre-existing infection at the block site, pre-existing chronic pain or cognitive dysfunction, and history of opioid abuse that would prevent patients from accurately participating in postoperative quality of recovery and analgesia assessment.

### Anesthesia Application

All patients were monitored in the operating room (OR) using standard ECG, non-invasive blood pressure, peripheral oxygen saturation, and dual frequency index (BIS). First, heart rate and mean arterial pressure (MAP) were measured as baseline (minute 0). After placing the 22-gage intravenous (IV) line, a 15 ml/kg/h isotonic saline IV infusion was performed among all patients under the same general anesthesia. Pre-oxygenation was employed to induce anesthesia for 3 min, and the intravenous injection of midazolam (0.05 mg/kg), sufentanil (0.5 μg/kg), propofol (2–3 mg/kg), and rocuronium (0.6 mg/kg) was made. The end-tidal carbon dioxide extent of 35–40 mmHg was kept with a double-lumen endotracheal catheter adopted for positive-pressure ventilation, and a fiber-optic bronchoscope was used to determine the correct location. During operation, the anesthesia maintenance regimen was propofol (50–150 μg kg^−1^ min^−1^) and remifentanil (0.1 μg^−1^ kg^−1^ min^−1^). An anesthesiologist was employed to titrate the minimum alveolar concentration of sevoflurane, and the BIS value of between 40 and 60 was kept. Volume control ventilation was applied with the coefficients below: tidal volume, 6–8 ml/kg; respiratory rate, 12–20 beats/min; and 2 L gas with 70% oxygen and 30% air.

During anesthesia, the intravenous administration of 0.1 μg/kg sufentanil was made when the heart rate or blood pressure was 20% higher than the basic value; the administration of 0.5 mg atropine was made when the heart rate was <50 beats/min, and the intravenous injection of ringer’s lactate solution of 250 ml or ephedrine of 0.1 mg kg^−1^ was made when the blood pressure was lower than 20% of the elementary value.

The administration of granisetron 3 mg was made 30 min before the surgery, so as to stop postoperative nausea and vomiting. All patients were sent to the postanesthesia care unit (PACU) after surgery. The administration of atropine of 0.01 mg/kg and neostigmine of 0.05 mg kg^−1^ was made to reverse the muscle relaxation role of rocuronium as required. The patients were sent to the surgery ward when they met the PACU discharging standard.

### Surgical Procedures

In patients with one trochlear port, a single 3.0–4.0 cm incision was made in the fourth or fifth intercostal space of the anterior axillary line and a trochal port was inserted on the chest wall; then, the surgery procedure was performed *via* the trochal port. A thoracic drainage tube was inserted through the incision before the skin of the fourth or fifth intercostal segments was closed.

### Patient Grouping and Randomization

Eligible patients were recruited by surgeons and research nurses. Patients were grouped into four, namely, group control (Group C), group R_0.2%_, R_0.3%_, and R_0.4%_, according to the ratio of 1:1:1:1 randomly. Random numbers were produced on computer and kept in sealed opaque envelopes. After the final part of the trial was randomized, the principal investigator (who would not receive any surgery) decided the four surgeons who would perform the surgery to balance the number of VATS steps for every surgeon. The main investigator or research nurse informed the surgeon of patient assignment the day before surgery and the operating room team on the day of surgery. After induction of anesthesia, RIB was done by an anesthesiologist who has experience in more than 30 cases of RIB independently. The researcher responsible for the 48-h postoperative follow-up was blind to the randomization group. In addition, during the preoperative follow-up, patients were instructed on how to apply a patient-stipulated intravenous analgesia (PCA) device for pain management and how to assess pain at rest and on movement applying the NRS scale.

### Application of Block Intervention

After induction of anesthesia, RIB was conducted according to the past description (Elsharkawy et al., 2016). A high-frequency linear ultrasonic probe (LOGIQ e ultrasound system, Deutschland GmbH and Co. KG, Solingen, Germany) was used. The medial placement of oblique sagittal plane was made on the medial margin of the scapula. Ultrasound identified trapezius, intercostal, rhomboid, pleura, and lung markers. Under aseptic conditions, the insertion of an 80-mm gauge 21 needle was conducted at the ultrasonic section T5–6. In the group R_0.2%_, the injection of a dose of 30 ml of 0.2% ropivacaine was performed in the fascial plane between the rhomboid and intercostal muscles; in the group R_0.3%_, the injection of a dose of 30 ml 0.3% ropivacaine was performed in the fascial plane between the rhomboid and intercostal muscles; and in the group R_0.4%_, the injection of a single dose of 30 ml 0.4% ropivacaine was performed in the fascial plane between the rhomboid and intercostal muscles. The diffusion of local anesthetic fluid under rhomboid muscle was observed by ultrasound.

### Analgesic Protocol and Assessment of Pain and Sensorial Block

In the PACU, all patients underwent patient-stipulated intravenous analgesia (PCIA): 150 μg sufentanil with a total of 150 ml, loading dose of 2 ml, background dose of 2 ml, and locking time of 15 min. Another blinded anesthesiologist made pain evaluation, about 30 min after being blocked with the 11-point NRS, ranging from 0 (no pain) to 10 (worst pain imaginable). In the surgical ward, the postoperative assessment of patients was made again at 0.5, 1, 3, 6, 12, 18, 24, 36, and 48 h. In case of the NRS mark of >3, the physician pressed the analgesia pump once and evaluate pain after 15 min. If the NRS mark was >3 continuously, the physician pressed the analgesia pump again. Rescue analgesia was made on basis of the anesthesiologist’s estimate with parecoxib sodium of 40 mg.

### Outcome Methods

This research held the main results of the overall QoR-40 scores 24 h after surgery between the four groups. There were a total of 40 questions for the assessment of five rehabilitation areas in this questionnaire: 12 items about physical comfort, 9 items about emotional state, 5 items about physical independence, 7 item about psychological support, and 7 item about pain (Kim et al., 2018). The secondary result methods were AUC of the NRS for pain at rest and on movement over 48 h, time of first postoperative analgesic request, postoperative 48-h opioid dosage, and satisfaction mark of patients (1–10, where 10 is the highest). Except these methods, dosage of propofol and remifentanil, PACU duration, postoperative nausea and vomiting (PONV), and total number of patients with a postoperative complication were put into record.

### Sample Size

The Power Exploration and Sample Size (PASS) 15.0 program (NCSS, LLC., Kaysville, UT, United States) was adopted to calculate the sample size of this research. Based on past researches, the 10-point diversities in QoR-40 marks between the group R_0.2%_ and group R_0.4%_ was considered clinically important (Myles et al., 2018; Altıparmak et al., 2020). On the basis of our preliminary research on 20 patients, the QoR-40 mark of group R_0.2%_ was 164.7 ± 5.5, and the QoR-40 mark of the group R_0.4%_ was 170.3 ± 5.9. Assuming α error = 0.05 (two-tailed), β error = 0.1 with a power of 0.90, at least 23 participants were required per group, considering the 20% dropout rate (on basis of a preliminary research); while increasing the sample scale, the research finally included 29 patients in every group.

### Statistical Exploration

SPSS version 25.0 (IBM Corp., Armonk, NY, United States) was adopted for data analysis. For every patient, the time interval with the NRS score was multiplied to calculate their AUC of NRS pain marks both on movement and at rest with GraphPad Prism version 7 (GraphPad Software Inc., San Diego, CA, United States). Continuous data were examined and tested for distribution with the Shapiro–Wilk test. One-way analysis of variance was adopted to explore normally distributed data for the comparison of group-wise diversities in the result coefficients [BMI, age, operation time, anesthesia time, remifentanil dosage, propofol dosage, preoperative QoR-40 mark, QoR-40 mark, physical comfort, emotional state, psychological support, physical independence, pain, the NRS mark curve (AUC) for pain at rest and on movement, time to first postoperative analgesic request, PACU duration, postoperative 48-h total amount of opioids, satisfaction score of patients, and parecoxib sodium for injections]. Normally distributed data are shown as mean ± standard deviation. The diversities among male/female, ASAI/II/III, total number of patients with a postoperative complication, operation procedure, and surgical incision (left chest/right chest) were compared with the chi-square test. Operation procedure and PONV scores were analyzed using Kruskal–Wallis test, and a 5-point numerical scale (0 = no symptom, 1 = scarcely, 2 = usually, 3 = most of the time, 4 = all the time) was adopted to assess PONV. *p*-values <0.05 were regarded significant for the test outcomes displayed.

## Results

**FIGURE 1:**
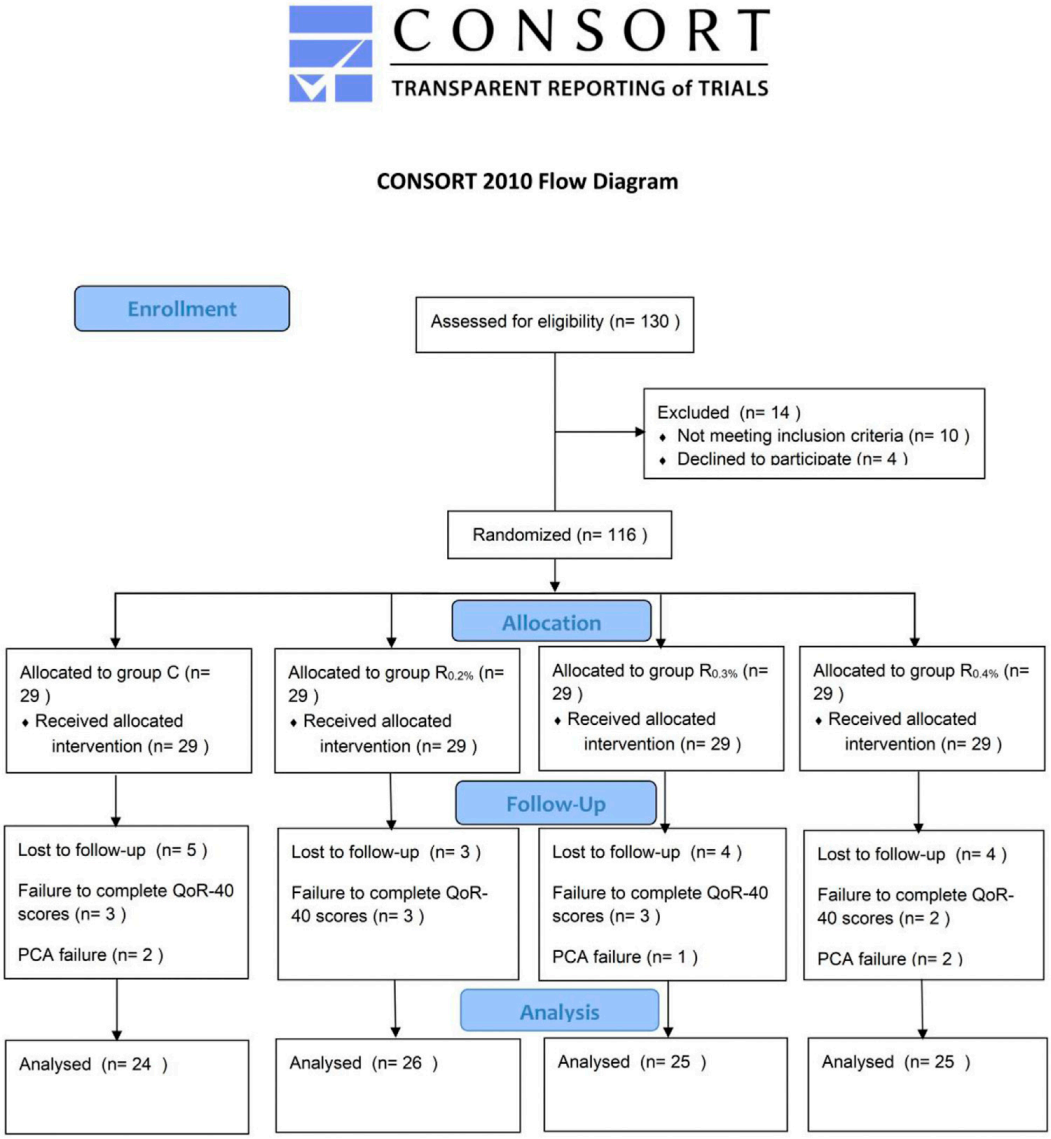
Consolidated standards of reporting trials statement flow diagram for the study.

The flowchart for reporting trials of the consolidated standards is shown in Figure 1. One hundred thirty patients were initially enrolled, out of which 10 patients did not satisfy the inclusion standards, four patients rejected to take part in, and 116 patients were eventually categorized into four groups. Five patients in the Group C were excluded due to uncompleted QoR-40 scores and PCA failure. Three patients in the group R_0.2%_ were excluded due to uncompleted QoR-40 scores. Four patients in the group R_0.3%_ were excluded because of uncompleted QoR-40 scores and PCA failure. Four patients in the group R_0.4%_ were excluded due to failure to complete QoR-40 scores and PCA failure. Therefore, 24 patients in the Group C, 26 patients in the group R_0.2%_, 25 patients in the group R_0.3%_, and 25 patients in the group R_0.4%_ were analyzed.

No diversities were observed in the baseline features between the groups (Table 1). QOR-40 scores are shown in Table 2. A great diversity was found between the mean global QoR-40 marks of the groups. Scores of all QoR-40 dimensions (except psychological support) varied statistically among four groups. Groups R_0.2%_, R_0.3%_, and R_0.4%_ displayed great diversities in the overall QoR-40 scores and QoR-40 dimensions (except psychological support) when compared with the Group C (*p* < 0.001 for all contrasts). Group R_0.3%_ and R_0.4%_ displayed great diversities in the overall QoR-40 scores and QoR-40 dimensions (except psychological support) when compared to the R_0.2%_ group (*p* < 0.001for all comparisons). The global QoR-40 scores (*p* = 0.054) and QoR-40 dimensions [physical comfort (*p* = 0.585), emotional status (*p* = 0.101), physical independence (*p* = 0.731), pain (*p* = 0.306)] did not vary greatly between the groups R_0.3%_ and R_0.4%_.

**TABLE 1:** Descriptive variable characteristics of patients in four groups ( $\bar{x}$ ± SD).

	Group C	Group R_0.2%_	Group R_0.3%_	Group R_0.4%_	*p* value
Sample size, n	24	26	*25*	25	
Age (years)	70.0 ± 4.7	66.6 ± 5.2	67.1 ± 5.1	68.3 ± 4.7	0.604^*^
Gender (male/female)	12/12	14/12	14/11	14/11	0.972^#^
BMI (kg/m^2^)	23.2 ± 3.4	24.1 ± 3.4	23.2 ± 2.6	22.7 ± 2.1	0.295^*^
Procedure duration (min)	109.1 ± 28.8	104.5 ± 21.7	105.9 ± 25.5	105.8 ± 26.0	0.608^*^
Duration of anesthesia (min)	130.6 ± 40.7	126.0 ± 23.2	127.2 ± 37.0	125.8 ± 30.2	0.062^*^
ASA class I/II/Ⅲ	2/18/4	3/19/4	2/20/3	3/19/3	0.994^#^
Surgical incision (left/right)	8/16	8/18	10/15	10/15	0.866^#^
Pre-QoR-40 score	182.4 ± 4.7	181.6 ± 3.9	181.3 ± 3.3	181.8 ± 3.8	0.804^*^
Operation procedure					0.996**
Wedge resection	15 (63%)	17 (65%)	14 (56%)	16 (64%)	—
Bullectomy	7 (29%)	8 (31%)	9 (36%)	8 (32%)	—
Lobectomy	2 (8%)	1 (4%)	2 (8%)	1 (4%)	—

Statistical tests: ^*^ *p* value is obtained with one-way analysis of variance. ^#^ *p* value is obtained with Pearson’s χ2 test. ^**^ *p* value is obtained with Kruskal-Wallis test.

**TABLE 2:** Global and dimension QoR-40 questionnaire score at 24th hour after operation in four groups ( $\bar{x}$ ±SD).

	Group C	Group R_0.2%_	Group R_0.3%_	Group R_0.4%_	*p* value
Sample size, n	24	26	*25*	25	
Global QoR-40 score	151.7 ± 3.8	164.3 ± 3.8	172.8 ± 3.4	174.6 ± 2.4	<0.001^*^
Physical comfort	43.8 ± 2.9	49.5 ± 1.9	50.7 ± 1.8	51.0 ± 1.5	<0.001^*^
Emotional status	34.3 ± 2.1	37.2 ± 1.5	38.9 ± 1.9	39.8 ± 1.7	<0.001^*^
Physical independence	19.8 ± 1.7	20.4 ± 2.3	21.6 ± 1.1	21.8 ± 1.1	<0.001^*^
Psychological support	29.9 ± 1.3	30.2 ± 1.4	30.4 ± 1.4	30.5 ± 1.3	0.440^*^
Pain	23.9 ± 1.6	27.1 ± 1.7	31.2 ± 1.1	31.6 ± 1.0	<0.001^*^

Statistical tests: Statistical tests: ^*^ *p* value is obtained with one-way analysis of variance.

The difference in NRS score AUC at rest and on movement in 48 h was statistically significant in both groups (Table 3). Groups R_0.2%_, R_0.3%_, and R_0.4%_ displayed great diversities in NRS score AUC at rest and on movement in 48 h when compared with the Group C (*p* < 0.001 for all contrasts). Group R_0.3%_ and R_0.4%_ displayed great diversities in NRS score AUC at rest and on movement in 48 h when compared with the R_0.2%_ group (*p* < 0.001 for all contrasts). The NRS score AUC at rest and on movement in 48 h did not vary greatly between the Group R_0.3%_ and R_0.4%_ (*p* > 0.05 for all contrasts).

**TABLE 3:** The AUC pain NRS vs time at rest and on movement of four groups ( $\bar{x}$ ± SD).

	Group C	Group R_0.2%_	Group R_0.3%_	Group R_0.4%_	*p* value
Sample size, n	24	26	*25*	*25*	
AUC pain NRS vs. time (at rest)					
0–6 h postoperatively	7.2 ± 1.4	4.9 ± 1.4	3.1 ± 1.6	2.4 ± 1.5	<0.001^*^
0–12 h postoperatively	23.4 ± 3.0	17.4 ± 2.3	12.9 ± 2.4	11.4 ± 2.9	<0.001^*^
0–24 h postoperatively	59.7 ± 5.2	45.8 ± 5.0	32.6 ± 4.7	29.8 ± 5.8	<0.001^*^
0–48 h postoperatively	116.9 ± 8.5	94.2 ± 8.0	70.4 ± 8.1	64.4 ± 10.3	<0.001^*^
AUC pain NRS vs. time (on movement)					
0–6 h postoperatively	17.3 ± 2.1	11.1 ± 1.4	8.8 ± 1.5	7.9 ± 1.4	<0.001^*^
0–12 h postoperatively	55.2 ± 3.5	40.3 ± 4.3	31.5 ± 2.7	29.9 ± 3.1	<0.001^*^
0–24 h postoperatively	119.9 ± 6.0	95.7 ± 7.4	74.5 ± 4.8	71.6 ± 5.4	<0.001^*^
0–48 h postoperatively	211.9 ± 11.6	170.7 ± 11.4	136.6 ± 7.5	129.7 ± 8.9	<0.001^*^

Statistical tests: ^*^ *p* value is obtained with one-way analysis of variance.

**FIGURE 2:**
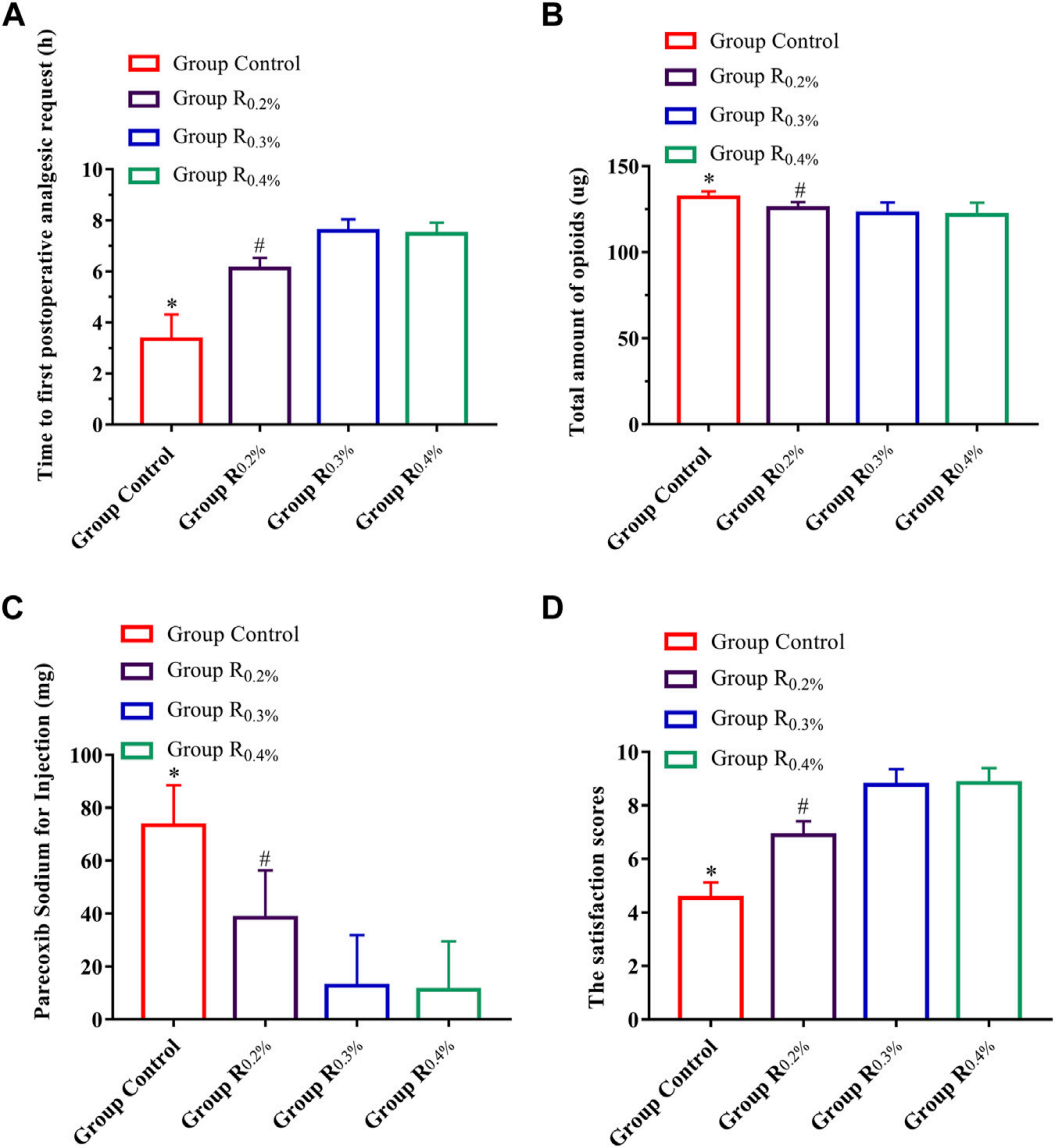
**(A)**. Time to first postoperative analgesic request, **(B)**. Postoperative 48-h total amount of opioids, **(C)**. Parecoxib sodium for injections, **(D)**. Satisfaction score of patients.^*^ *p* < 0.05 compared with R_0.2%_, R_0.3%_, and R_0.4%_ groups, ^#^ *p* < 0.05 compared with R_0.3%_ and R_0.4%_ groups.

Time of first postoperative analgesic request, postoperative 48-h total amount of opioids, parecoxib sodium for injections, and satisfaction score of patients in the groups R_0.2%_, R_0.3%_, and R_0.4%_ displayed great diversities when compared with the group C (*p* < 0.001 for all contrasts) (Figures 2A–D), and time to first postoperative analgesic request, postoperative 48-h total amount of opioids, parecoxib sodium for injections, and satisfaction score of patients in the R_0.3%_ and R_0.4%_ also displayed great diversities when compared with Group R_0.2%_, (*p* < 0.001 for all contrasts) (Figures 2A–D). The time to first postoperative analgesic request (*p* = 0.5), postoperative 48-h total amount of opioids (*p* = 0.526), parecoxib sodium for injections (*p* = 0.750), and satisfaction score of patients (*p* = 0.671) did not vary greatly between the groups R_0.3%_ and R_0.4%_.

Groups R_0.2%_, R_0.3%_, and R_0.4%_ displayed great diversities in the dose of propofol and remifentanil applied and recovery room duration when compared to the Group C (*p* < 0.001 for all contrasts) (Table 4). Groups R_0.3%_ and R_0.4%_ displayed great diversities in the dose of propofol and remifentanil applied and recovery room duration when compared to the R_0.2%_ group (*p* < 0.001 for all comparisons). The dose of propofol (*p* = 0.562) and remifentanil (*p* = 0.498) used and recovery room duration (*p* = 0.664) did not vary greatly between the groups R_0.3%_ and R_0.4%_. No great diversities were shown in PONV scores (*p* = 0.851 for all contrasts) and total number of patients with a postoperative complication (*p* = 0.924 for all contrasts) among the four groups (Table 5).

**TABLE 4:** Intraoperative anesthetic dosage, postoperative analgesic, and recovery of four groups ( $\bar{x}$ ± SD).

	Group C	Group R_0.2%_	Group R_0.3%_	Group R_0.4%_	*p*-value
Sample size, n	24	26	*25*	25	
Remifentanil (µg)	463.8 ± 77.2	415.4 ± 52.8	353.6 ± 64.9	341.2 ± 61.8	<0.001^*^
Propofol (mg)	477.9 ± 78	421.9 ± 59.7	341.2 ± 74.9	329.2 ± 78.0	<0.001^*^
PACU duration (min)	22.4 ± 5.0	18.2 ± 2.5	15.6 ± 1.3	15.3 ± 1.3	<0.001^*^
PONV scores, n (%)					0.851^**^
0	12	15	17	18	
1	4	7	8	7	
2	5	3	0	0	
3	2	1	0	0	
4	1	0	0	0	

PONV was assessed using a 5-point numerical scale (0 = no symptom, 1 = scarcely, 2 = usually, 3 = most of the time, 4 = all the time).

^*^ *p* value is obtained with one-way analysis of variance.

^#^ *p* value is obtained with Pearson’s χ^2^ test.

^**^ *p* PONV scores were analyzed using Kruskal–Wallis test

**TABLE 5:** Postoperative complications in four groups ( $\bar{x}$ ± SD).

	Group C	Group R_0.2%_	Group R_0.3%_	Group R_0.4%_	*p*-value
Sample size, n	24	26	*25*	25	
Total number of patients with a postoperative complication	3	2	2	2	0.924^#^
Postoperative pneumonia	0	1	1	1	
Surgical site infection	0	0	0	0	
Recurrent pneumothorax/air leak requiring further intervention	1	0	0	0	
Arrhythmia	1	0	0	0	
Bleeding requiring transfusion	1	1	1	2	
Unplanned ICU admission	0	0	0	0	
Acute kidney injury	0	0	0	0	

Statistical tests: ^#^ *p* value is obtained with Pearson’s χ^2^ test.

## Discussion

This is the first randomized, double-blind clinical trial that compared different concentrations of ropivacaine RIB block in VATS with the patient-centered result method, QoR-40. A clinically meaningful improvement was shown in QoR at 24 h for patients who underwent 0.3% ropivacaine RIB in comparison with a 0.2% ropivacaine RIB. Furthermore, 0.3% ropivacaine RIB had a smaller burden of pain over time (AUC of NRS) at rest and on movement and less total amount of opioids. The highest concentration of ropivacaine (0.4%) did not show a great merit in terms of postoperative analgesia applying the RIB.

A current international movement uses more patient-centered results in assessing the effectiveness of anesthetic interventions. While lower pain marks are significant, patients may not consider it an excellent recovery experience with other debilitating side effects. The QoR-40 scores are internationally recognized as a valid method to assess patients’ quality of recovery after surgery (Kim et al., 2018).

To the best of our ability to review the literature, this trial is the first to assess the effectiveness of various concentrations of ropivacaine RIB in postoperative analgesia in thoracic surgery using QoR-40. Deng et al. (2020) found that 0.3% ropivacaine was the best concentration for pectoral nerve block type II (PECS II block) among patients who have received modified radical mastectomy (MRM) for breast cancer and that a 0.3% concentration provided effective analgesia for MRM for 48 h. Increasing the concentration of ropivacaine did not significantly enhance the analgesic effect of the PECS II block. Su et al. (2015) found that in ultrasound-guided regional anesthesia, growing the concentration of ropivacaine at the same volume led to a progressive growth in analgesia, and 0.4% ropivacaine was not superior to 0.3% ropivacaine in terms of analgesia. At the same time, increasing the blood concentration of ropivacaine may increase the risk of neurotoxicity. Some investigators have applied *in vivo* or *ex vivo* animal models to study the neurotoxicity of local anesthetics, and both showed a concentration-dependent effect on the degree of nerve damage, with higher concentrations resulting in more severe damage. However, it remains to be further studied clinically as to which concentrations are also associated with which adverse effects. In the present experiment, no serious adverse reactions were observed with RIB blockade at 0.2%, 0.3%, and 0.4% concentrations of ropivacaine (Brull et al., 2007).

Our findings, by comparing the QoR-40 scores of 0.2%, 0.3%, and 0.4% ropivacaine RIB, were similar to theirs, but their assessed result was confined to pain severity and time to postoperative opioid demands. Our study showed that improvement of QoR-40 scores after VATS by RIB with ropivacaine relied on the concentration of ropivacaine; 0.2% ropivacaine did not improve QoR-40 scores after VATS, and 0.3% ropivacaine RIB improved QoR-40 scores significantly after VATS in patients, but when the concentration of ropivacaine was increased to 0.4% VATS, there was no great change in QoR-40 scores in patients after surgery.

Most of the existing methods focus on minimizing the loss of sample pairs. However, in many applications, the number of intra- and interclass sample pairs may be highly unbalanced, which may lead to deteriorating or suboptimal performance, and for such unbalanced distribution problems, AUC can be considered as a more meaningful performance metric (Huo et al., 2018). Therefore, in this study, we used AUC to count NRS scores over 48 h. Then, we found that the AUC of NRS in 48 h was significantly improved with 0.3% ropivacaine as compared to 0.2% ropivacaine; however, no great change was found in the AUC of NRS when it was increased to 0.4%. In addition, time of first postoperative analgesic request, recovery time, postoperative 48-h opioid dosage, and satisfaction score of patients can also be proved. At 0.2% ropivacaine RIB, the patient’s time to first postoperative analgesic request is short, postoperative 48-h opioid dosage is large, the injection amount of parecoxib sodium is large, and the patient satisfaction is also low. When the ropivacaine concentration grew to 0.3%, there was a significant improvement, but when the ropivacaine concentration grew to 0.4%, there was no significant change.

It is also important to note that in this trial, when comparing the AUC of NRS scores at 0–48 h between the four groups, in order to compensate for the true number of patients in Group C, Group R_0.3%_, and Group R_0.4%_, we used the mean of the NRS in each group to replace the number of patients missing, with two patients missing in Group C and one patient each in Group R_0.3%_ and Group R_0.4%_.

Our study has some restrictions. First, a sham block was not given to the control group due to the ethical considerations of making an injection with no administration of a therapeutic drug. Second, no concentration gradient was used to decrease the number of groups and false-negative outcomes from various comparisons; ropivacaine studies at higher concentrations were not performed. Third, there was no measurement of plasma ropivacaine levels at various concentrations; although past studies have not reported any RIB-related adverse reactions, pharmacokinetic studies were not performed.

## Conclusion

In this study, it was found that a dose of 0.3% ropivacaine is the best concentration for the RIB for patients who have received VATS. Through increasing ropivacaine concentration, the analgesia of the RIB was not improved greatly.

## Data Availability

The raw data supporting the conclusion of this article will be made available by the authors, without undue reservation.
